# Women’s sexual health six months after a severe maternal morbidity event*

**DOI:** 10.1590/1518-8345.3500.3293

**Published:** 2020-06-19

**Authors:** Lisiane Camargo Alves, Jessica Ribeiro Costa, Juliana Cristina dos Santos Monteiro, Flávia Azevedo Gomes-Sponholz

**Affiliations:** 1 Universidade de São Paulo, Escola de Enfermagem de Ribeirão Preto, PAHO/WHO Collaborating Centre at the Nursing Research Development, Ribeirão Preto, SP, Brazil.; 2 Scholarship holder at the Coordenação de Aperfeiçoamento de Pessoal de Nível Superior (CAPES), Brazil.

**Keywords:** Morbidity, Sexuality, Pregnancy Complications, Pregnancy, Postpartum Period, Nursing, Morbidade, Sexualidade, Complicações na Gravidez, Gravidez, Período Pós-Parto, Enfermagem, Morbilidad, Sexualidad, Complicaciones del Embarazo, Embarazo, Periodo Posparto, Enfermería

## Abstract

**Objective:**

to investigate female sexual function in women six months postpartum and to compare sexual function among women who had and who did not have severe maternal morbidity (SMM).

**Method:**

a cross-sectional study conducted with 110 women in the postpartum period, with and without SMM. Two instruments were used, one for the characterization of sociodemographic and obstetric variables and the Female Sexual Function Index (FSFI) for sexual function. Univariate, bivariate and regression model analyses were performed.

**Results:**

FSFI scores showed 44.5% of female sexual dysfunction, of which 48.7% were among women who had SMM and 42.0% among those who had not. There were significant differences between age (*P*=0.013) and duration of pregnancy (*P*<0.001) between women with or without SMM. Among the cases of SMM, hypertensive disorders were the most frequent (83%). An association was obtained between some domains of the FSFI and the following variables: orgasm and self-reported skin color, satisfaction and length of relationship, and pain and SMM.

**Conclusion:**

white women have greater difficulty in reaching orgasm when compared to non-white women and women with more than 120 months of relationship feel more dissatisfied with sexual health than women with less time in a relationship. Women who have had some type of SMM have more dyspareunia when compared to women who have not had SMM.

## Abstract (pt)

**Objetivo:**

investigar a função sexual feminina em mulheres seis meses pós-parto e comparar a função sexual entre mulheres que tiveram e que não tiveram morbidade materna grave (MMG).

**Método:**

estudo transversal, com 110 mulheres no período pós-parto, com e sem MMG. Foram utilizados dois instrumentos, um para caracterização das variáveis sociodemográficas e obstétricas e o Female Sexual Function Index (FSFI) para a função sexual. Foram realizadas análises univariada, bivariada e modelo de regressão.

**Resultados:**

os escores do FSFI mostraram 44,5% de disfunção sexual feminina, sendo 48,7% entre as mulheres que tiveram MMG e 42,0% entre as que não tiveram. Houve diferenças significativas entre idade (*P*=0,013) e duração da gravidez (*P*<0,001) entre as mulheres com ou sem MMG. Dentre os casos de MMG, os distúrbios hipertensivos foram os mais frequentes (83%). Obteve-se associação entre alguns domínios do FSFI e as variáveis: orgasmo e cor autorreferida, satisfação e tempo de relacionamento, e dor e MMG.

**Conclusão:**

mulheres brancas têm maior dificuldade de atingir orgasmo quando comparadas com mulheres não-brancas e mulheres com mais de 120 meses de relacionamento sentem-se mais insatisfeitas com a saúde sexual do que mulheres com menor tempo de relacionamento. Mulheres que tiveram algum tipo de MMG apresentam mais dispareunia quando comparadas com mulheres que não tiveram MMG.

## Abstract (es)

**Objetivo:**

investigar la función sexual femenina en las mujeres seis meses después del parto y comparar la función sexual entre las mujeres que tenían y que no tenían morbilidad materna extrema (MME).

**Método:**

estudio transversal con 110 mujeres en el posparto, con y sin MME. Se utilizaron dos instrumentos, uno para la caracterización de variables sociodemográficas y obstétricas y el Índice de *Female Sexual Function Index* (FSFI) para la función sexual. Se realizaron análisis de modelos univariados, bivariados y modelo de regresión.

**Resultados:**

los puntajes del FSFI mostraron el 44.5% de la disfunción sexual femenina, de los cuales el 48.7% correspondió a mujeres que tenían MME y el 42% a las que no. Hubo diferencias significativas entre la edad (P = 0.013) y la duración del embarazo (P <0.001) entre mujeres con o sin MME. Entre los casos de MME, los trastornos hipertensivos fueron los más frecuentes (83%). Se obtuvo una asociación entre algunos dominios del FSFI y las variables: orgasmo y color autoinformado, satisfacción y duración de la relación, y dolor y MME.

**Conclusión:**

las mujeres blancas tienen mayor dificultad para alcanzar el orgasmo si se las compara con las mujeres no blancas y las mujeres con más de 120 meses de relación se sienten más insatisfechas con la salud sexual que las mujeres con menos tiempo de relación. Las mujeres que han tenido algún tipo de MME tienen más dispareunia en comparación con las mujeres que no han tenido MME.

## Introduction

Severe Maternal Morbidity (SMM) is characterized as an event of severe morbidity in women during pregnancy, childbirth, or 42 days postpartum. Classified as a potential life-threatening condition, it is defined by some specific criteria, such as hemorrhagic disorders; hypertensive disorders; other systemic diseases, such as endometritis, pulmonary edema; and some severity indicators such as blood transfusion and admission to the Intensive Care Unit (ICU)^(^ 1 ^)^.

SMM is currently a development indicator and, as it is more prevalent than maternal mortality, its monitoring is a strategy for preventing and combating maternal mortality^(^ 2 ^)^. Similar behavior to the maternal mortality ratio, SMM is higher in low- and middle-income countries when compared to high income countries^(^ 3 ^)^.

However, the magnitude of SMM is unknown; it is estimated that its occurrence is increasing over the years and that there are 20 to 30 SMM events for each maternal death^(^ 4 ^)^. These events, whether acute or chronic, cause sequelae that can compromise life activities prior to obstetric complications. Recent data estimates 27 million SMM episodes annually worldwide^(^ 5 ^)^.

The results of research using the criteria established by the WHO point to Africa as the continent with the highest global prevalence of SMM^(^ 5 ^)^, whose ratio ranges from 8/1000 live births (LBs) in Rwanda^(^ 6 ^)^ to 88.6/1000lb in Somalia^(^ 7 ^)^. In Asia, SMM ranges from 3.8/1000 LBs in Nepal^(^ 8 ^)^ to 120/1000 LBs in India^(^ 9 ^)^. Latin America and the Caribbean have the most disparate data on the world scenery, with the lowest SMM rate in Argentina being 2.62/1000 LBs and the highest in Peru at 34.92/1000 LBs^(^ 10 ^)^. Brazil is in a median position, with a prevalence of 10.21/1000 LBs^(^ 11 ^)^. In developed countries, the studies found vary in method and definition used; however, these have the lowest data in the world, ranging from 3/1000 LBs in Ireland to 7.3/1000 LBs in the United States^(^ 5 ^)^.

Although several studies have been developed on how SMM affects women’s quality of life^(^ 10 ^)^, we find few studies in our search on the consequences of SMM on the sexual health of surviving women^(^ 12 ^-^ 13 ^)^.

A study carried out in Asia with women who had SMM showed that most of them had difficulty in having orgasms and had reports of pain during sexual intercourse^(^ 14 ^)^. Another study, conducted in Africa, found a prevalence of dyspareunia among women exposed or not to SMM but there were no significant differences^(^ 13 ^)^. Based on the long-term consequences of SMM on women’s health, it is questioned in this article, whether women who have had SMM have more somatic complaints related to sexual function than women who have not had an episode of SMM.

## Method

This is an observational epidemiological study with a cross-sectional design, conducted with 110 women, recruited concurrently in two health services: Reference Center for Women’s Health in Ribeirão Preto (*Centro de Referência da Saúde da Mulher de Ribeirão Preto*, CRSMRP-MATER) and Clinical Hospital, Medical School of Ribeirão Preto, University of São Paulo (*Hospital das Clínicas da Faculdade de Medicina de Ribeirão Preto da Universidade de São Paulo*, HCFMRP/USP). Subsequently, visits were made to the participants’ homes for data collection.

Ribeirão Preto is home to the 13^th^ Regional Health Department (*Departamento Regional de Saúde*, DRS XIII) of the state of São Paulo, a regional health reference for 26 municipalities, covering an estimated population of 1,300,000 inhabitants. In this context, CRSMRP-MATER is a regional reference for gynecological and obstetric cases of medium complexity and performs about one third of usual risk and medium risk births of the Brazilian Public Health System (*Sistema Único de Saúde*, SUS). In this institution, participants were recruited who did not suffer injuries during pregnancy, delivery or immediate postpartum. HCFMRP/USP is a tertiary care hospital, a regional reference center for health, serving the municipalities of DRS XIII, other health departments and other states in Brazil. In this institution, participants who had an episode of SMM were recruited.

The reference population consisted of women in the immediate postpartum period, admitted to the wards of joint accommodation of both health services. The participants were selected according to the following inclusion criteria: postpartum women in hospital discharge process, over 18 years of age, residing in Ribeirão Preto or municipalities up to 60 kilometers away. This last inclusion criterion was established by the need for budgetary restrictions on research for trips longer than 60 kilometers. In the case of women recruited at HCFMRP/USP, those who met the SMM criteria established in this study were included, which followed the WHO classification. Exclusion criteria were women who use drugs or other psychoactive substances, with a history of psychiatric illness or chronic illnesses, such as cancer, diabetes and neurological diseases, as these factors negatively interfere in one or more phases of the sexual response^(^ 15 ^)^.

A simple random probabilistic sample was calculated based on the prevalence of SMM at HCFMRP/USP in the year prior to data collection. The tolerable figures were a sample error of 5%, a confidence level of 95%, and an expected loss of 10% for women who had SMM and those who did not have SMM. The sample calculation indicated the need for 231 participants, 77 of whom were recruited from HCFMRP/USP and 154 from CRSM-MATER.

The recruitment of participants took place from daily trips, on working days, to the joint accommodation units of HCFMRP/USP at 1 pm and CRSM-MATER from 4 pm. Through consultation with the hospital nursing census, women who were discharged home were identified and, using the established criteria, were invited to participate in the research. All of them were informed about the interview that would take place at home 180 days after hospital discharge, about the collection of data in the medical record and other aspects of the research. After being aware of the research and ethical aspects, the women who agreed to participate signed the Free and Informed Consent Form (FICF). At this time, data was collected from medical records. Data collection took place from May 2015 to August 2017.

For data collection, two structured instruments were used. The first instrument included the search for identification data, sociodemographic, obstetric, neonatal and SMM characteristics in the case of women recruited at HCFMRP/USP. This instrument was used both in the collection of medical record data (SMM data, obstetric and neonatal variables) and in the interview (sociodemographic and gynecological data). The second data collection instrument was the Female Sexual Function Index (FSFI), a questionnaire that evaluates female sexual health, applied in the home interview, 180 days after discharge. This instrument was developed in the United States, validated and adapted for Brazil^(^ 16 ^)^. The questionnaire contains 19 questions, which assess the sexual activity in the last four weeks, divided into six domains: desire, arousal, lubrication, orgasm, satisfaction and pain, where each one has a score and the total score refers to the sum of the scores multiplied by their respective factor. If the total value is less than or equal to 26.55, it indicates that the participant has some type of sexual dysfunction^(^ 16 ^)^.

The data were stored in a spreadsheet structured in Microsoft Excel, with double entry to validate the data entered and ensure reliability in the compilation. To verify the association between the variables, the *Mann-Whitney and Fisher’s Exact* tests were used. In all the tests, p values <0.05 were considered statistically significant. To assess the adequacy of the selected response variable, the Shapiro-Wilk Normality test was applied to the residuals of the adjustment. Data analysis was performed using the R free version 3.4.3 software, with a significance level of 5% (α = 0.05).

The study was approved by the Research Ethics Committee linked to the National Research Ethics Committee of the National Health Council with the CAAE protocol: No. 37254814.7.0000.5393.

The criteria for the characterization of SMM were the following: hemorrhagic disorders (placental abruption, accrete/increte/percrete placenta, ectopic pregnancy, antepartum and/or postpartum hemorrhage, uterine rupture, abortion with severe hemorrhage); hypertensive disorders (severe pre-eclampsia, eclampsia, severe hypertension, hypertensive encephalopathy, HELLP syndrome); other systemic diseases (endometritis, pulmonary edema, respiratory failure, seizures, sepsis, thrombocytopenia <100,000, thyroid crisis); severity management indicators (blood transfusion, central venous access, hysterectomy, ICU admission, prolonged hospital stay [> 7 days postpartum], intubation no related to the anesthetic procedure, return to the operating room, major surgical interventions)^(^ 1 ^)^.

## Results

Of the 110 women who participated in the study, 41 had SMM and 69 did not have any problems during the pregnant-puerperal cycle. Table 1 shows the sociodemographic characteristics and reproductive history of women with and without SMM.

**Table 1:** Sociodemographic characteristics and reproductive history of participants with and without SMM*

Variables	SMM* (n=41)		*(n=69)		*P* value^‡^
	Mean(SD^†)^	n (%)	Mean (SD^†^)	n (%)	
Sociodemographic characteristics					
Age (years)	30.0 (5.5)		27.1 (5.2)		0.013
Family income (month)					0.169
≤ 1 minimum wage^§|^		12 (29.3)		19 (27.5)	
2-3 minimum wages^§^		19 (46.4)		30 (43.5)	
≥ 4 minimum wages^§^		8 (19.5)		4 (5.8)	
Do not know		2 (4.8)		16 (23.2)	
Self-referred color					0.175
White		19 (46.3)		23 (33.3)	
Non-white		22 (53.7)		46 (66.7)	
Schooling (years)	9.5 (2.8)			9.7 (2.3)	
1 - 8		12 (29.3)		19 (27.5)	
9 - 11		26 (63.4)		46 (66.6)	
≥12		3 (7.3)		4 (5.8)	
Occupation					
Unemployed		12 (29.3)		15 (21.7)	
Housewife		12 (29.3)		37 (53.6)	
Paid work		17 (41.5)		17 (24.6)	
Reproductive history					
Pregnancies	2.4 (1.5)		2.4 (1.4)		0.763
Primiparity		13 (31.7)		20 (28.9)	
Multiparity		28 (68.3)		49 (71.1)	
Gestation time					
Term (≥ 37 s)		26 (63.4)		64 (92.8)	<0,001
Preterm (<37s)		15 (36.6)		5 (7.2)	
Type of delivery					0.076
Vaginal		17 (41.4)		42 (60.9)	
C-Section		24 (58.6)		27 (39.1)	
MAC usage^‖^ (last year)					
Yes		29 (97.6)		69 (100)	
No		12 (2.4)		-	

* SMM = Severe Maternal Morbidity;

† Standard Deviation;

‡ Fisher's exact test ;

§ Minimum wage 2016 = R$ 880.00;

‖ Contraceptive method

Among the sociodemographic characteristics, there was a statistical difference only between the age of the women (mean 30.0 years old; SD 5.5) versus (mean 27.1 years old; SD 5.2); *P* = 0.013. As for the obstetric aspects, the mean of pregnancies was 2.4 with a standard deviation of 1.5 for women who had SMM and 1.4 for those who did not, and a median of 2.0 pregnancies. All the participants underwent prenatal care and met the recommendations of the Ministry of Health for a minimum of six consultations. Regarding the type of delivery, 58.6% of the women with SMM underwent c-section and 60.4% of the women who did not have SMM had a normal delivery. Women who had SMM were more likely to have preterm births (63.4% vs 92.8%; *P* <0.001).

Among the cases of SMM, hypertensive disorders were the most frequent (83%), with severe hypertension and severe pre-eclampsia, the most incident diagnoses (Table 2).

**Table 2:** Distribution of SMM* among the study participants

SMM type*	Frequency	Percentage
Hemorrhagic disorders	3	7.3
Ablatio Placentae	2	66.7
Antepartum hemorrhage	1	33.3
Hypertensive disorders	34	83.0
Severe pre-eclampsia	13	38.2
Eclampsia	2	5.9
Severe Hypertension	16	47.1
HELLP syndrome^†^	3	8.8
Systemic diseases	4	9.7
Convulsions	2	50.0
Thyroid complications	2	50.0

* SMM = Severe Maternal Morbidity;

† HELLP = Hemolysis Elevated Liver enzymes Low Platelet count

Table 3 shows the comparison of each domain and the total FSFI score between women with and without SMM.

**Table 3:** Comparison of the domains and scores of sexual function according to FSFI between women with and without SMM*

Sexual function	SMM (n=41)	Without SMM (n=69)	*P*-value
	Mean (SD^†^)	Mean (SD^†^)	
Desire	3.32 (1.48)	3.88 (1.18)	0.051
Arousal	3.65 (1.41)	3.99 (1.16)	0.273
Lubrication	4.47 (1.54)	4.95 (1.33)	0.100
Orgasm	4.24 (1.65)	4.28 (1.44)	0.921
Satisfaction	4.48 (1.44)	4.99 (1.00)	0.131
Pain	4.84 (1.63)	4.81 (1.35)	0.161
Total	25.03 (6.91)	26.93 (6.03)	0.243

* SMM = Severe Maternal Morbidity;

† SD = Standard Deviation; ^‡^p value = Mann-Whitney test

The prevalence of female sexual dysfunction was 44.5% (FSFI score ≤ 26.55), with 48.7% among women who had SMM and 42.0% among those who did not have SMM.

From the analysis of the data of the participants in this study, no association was found between the occurrence of female sexual dysfunction and SMM.

When analyzing the domains that make up the FSFI, there was an association between the presence of SMM and pain during sexual intercourse, regardless of age, length of relationship, income, self-reported skin color, parity and type of delivery (Table 4).

**Table 4:** Association between the orgasm, satisfaction and pain during sexual intercourse domains and the self-reported skin color, relationship time and SMM variables, in an Inflated Beta regression model

Variables	Orgasm		
	Mean	P value	CI* 95.0%
Self-reported color			
White	0.5942	0.01	0.5095-0.6737
Non-white	0.7020		0.5390-0.8259
	**Satisfaction**		
Relationship time			
≥ 120 months	0.5934	0.03	0.6188-0.7768
< 60 months	0.7039		0.3899-0.7692
	**Pain in intercourse**		
SMM^†^	0.6665	0.002	0.4995-0.8000
Without SMM^†^	0.7973		0.5607-0.9238

* CI = Confidence Interval;

† SMM = Severe Maternal Morbidity

The women who reported being non-white performed better in sexual response in the orgasm domain of FSFI than women who reported being white (*P*= 0.01). Women with more than 120 months of relationship felt more dissatisfied with their sexuality than women with a relationship of up to 60 months (*P*= 0.03). Women who had SMM reported more pain during intercourse than women who did not have SMM (*P*= 0.002).

## Discussion

In this study, there were statistically significant differences between the age (*P*= 0.013) and duration of pregnancy (P<0.001) variables between women with or without SMM. However, in addition to these two variables, the characterization of these women shows similarities with several studies, both in our country and abroad. These data agree with a study carried out in the state of Sergipe^(^ 17 ^)^, where the authors found the occurrence of SMM among women over 35 years old (p=0.038) when compared with younger women with SMM. Also corroborating this result we find a study carried out in Asia^(^ 14 ^)^, whose women who had SMM were significantly older than women who did not have SMM. SMM (31.6 years old;

SD=6.26) versus (29.2 years old; SD=5.65) *P*<0.001. Severe maternal morbidity puts at risk not only the woman’s life, but of the fetus/newborn, when prematurity and its consequences occur^(^ 3 ^)^. In our study, preterm delivery was associated with SMM, as well as studies developed in Finland^(^ 18 ^)^and in Brazil^(^ 19 ^)^.

Among the cases of SMM, hypertensive disorders were the most frequent in the present study, characterized by severe hypertension and severe pre-eclampsia. A review study on women with hypertensive crises in pregnancy showed that hypertension is one of the complications that appears most in clinical practice, being in first place among the causes of maternal deaths. Arterial hypertension complicates 7 to 10% of all the pregnancies, highlighting the need for strategies and interventions to prevent and qualify the health system, where pregnant women need distinct prenatal care, with a more careful assessment, specific exams and actions aimed at reducing maternal mortality and severe morbidity^(^ 20 ^)^. In view of the results of the present study, it was shown that among hypertensive disorders, 39% of women had severe hypertension and 43.9% were diagnosed with severe pre-eclampsia, eclampsia and HELLP syndrome, diseases that can be prevented by sensitizing pregnant women and professionals regarding the importance well-performed prenatal care.

With regard to female sexual function, the present study showed that 44.5% of the women had sexual dysfunction. Although this study did not find a significant association between SMM and sexual dysfunction, the mean score obtained by FSFI characterized sexual dysfunction among women with SMM. A study carried out in Campinas, SP, compared these two groups of women, using FSFI, and found no association between groups; however, the score obtained indicated dysfunction both in the group of women with morbidity and in the control group^(^ 13 ^)^.

From the Inflated Beta regression analysis, the present study found a significant association between some FSFI domains and some women’s variables: orgasm and self-reported skin color, satisfaction and length of relationship, and pain and SMM, corroborating data from a study that found association between sexual dysfunction and skin color, demonstrating that non-white women find it easier to reach orgasm when compared to white women^(^ 12 ^)^. With regard to dyspareunia, our study found that women who suffered some type of injury during pregnancy, childbirth or postpartum had higher rates of pain during sexual intercourse when compared to women who went through a pregnancy without any type of injury. This result agrees with a research carried out in Campinas which revealed that maternal morbidity is directly associated with a higher frequency of dyspareunia after delivery^(^ 15 ^)^.

As for the limitations of this study, it is considered that, as it is a cross-sectional study, a characteristic limitation is the fact that the data are collected at only one point in the lives of these women. Thus, there is no way to state that women who scored less than 26.55 did not previously have any type of sexual dysfunction. In a prospective cohort study, data would be collected at various times and there would be a follow-up of the sexual life of these women. In this context, the importance of the nurse’s approach to the topic in women’s routine consultations is emphasized. It must be considered that sexuality is still a subject whose approach generates discomfort. In this sense, despite having their privacy preserved, many women may have felt shy at the time of the interview, which may have interfered with the results. Another limitation lies in the fact that the instrument used was designed to be applied to women without any type of health problem. As far as we know, there is no specific instrument in the literature to assess the sexual health of women who have had an event of severe maternal morbidity.

## Conclusion

Our study showed that white women have greater difficulty in reaching orgasm when compared to non-white women and that women with more than 120 months of relationship feel more dissatisfied with sexual health than women with less time in relationship.

However, women who had some type of SMM have more dyspareunia when compared to women who did not have any type of injury during pregnancy, childbirth or postpartum.

We understand that these findings show that women’s sexual health should be better assessed in routine consultations and that SMM may have an influence on the sexual response after delivery. Thus, we hope that our results will stimulate further studies with a view to a better approach to female sexual health and the prevention of problems during pregnancy, childbirth or postpartum.
